# Management of Femoral Condyle Insufficiency Fractures Following Total Knee Arthroplasty (FCIF-TKA)

**DOI:** 10.7759/cureus.75832

**Published:** 2024-12-16

**Authors:** Walter-Soon-Yaw Wong, Haobin Chen, Joel W Lim, Wei Ming Siow, Kein Boon Poon

**Affiliations:** 1 Orthopedic Surgery, Sengkang General Hospital, Singapore, SGP

**Keywords:** femoral condyle insufficiency, fracture, periprosthetic, posterior stabilized, total knee arthroplasty

## Abstract

Background: Femoral condyle insufficiency fractures following total knee arthroplasty (FCIF-TKA) are rare but significant complications. These fractures, characterized by atraumatic bone insufficiency near the femoral component, present unique challenges in postoperative care, often necessitating femoral component revision.

Methods: This study retrospectively reviewed 835 primary total knee arthroplasties performed by a single surgeon, identifying six cases of FCIF-TKA. Patient demographics, implant details, radiographic findings, and clinical outcomes were analyzed with up to four years of follow-up. The study aimed to identify risk factors, evaluate management outcomes, and assess pain and mobility improvements.

Results: The incidence of FCIF-TKA was 0.72%, notably higher than previously reported. All cases occurred in female patients with varus deformity, a mean age of 72 years, and a mean BMI of 33.48. Bone mineral density scans revealed osteopenia or osteoporosis in most cases. All patients had stemless posterior-stabilized (PS) femoral implants, with a mean diagnosis time of 17.2 days post-surgery. Conservative management using a hinged knee brace and a 12-week protected weight-bearing protocol proved effective for compliant patients. VAS scores improved from 4.8 at diagnosis to 2.0 at six months and 0.67 at one year. ROM increased from 76.3° postoperatively to 94.2° in one year. One non-compliant patient required revision due to fracture progression. At four years, 83.3% of cases showed implant survivorship without further revision.

Conclusions: FCIF-TKA appears associated with advanced age, female sex, high BMI, osteoporosis, and severe varus deformities, particularly with stemless PS femoral implants. Preventive strategies may include using cruciate retaining implants and femoral stems to increase the metal-bone surface area over weight-bearing portions of the femoral implant. Conservative management with a hinged knee brace and structured weight-bearing for 12 weeks yields satisfactory pain and ROM improvements at one year, with high survivorship at four years, indicating that immediate revision may not be necessary for all patients.

## Introduction

Femoral condyle insufficiency fractures following total knee arthroplasty (FCIF-TKA) are a serious but rare complication, with a reported prevalence of 0.05% and fewer than 50 cases documented in the published literature [1]. Although technically categorized as a type of periprosthetic fracture, FCIF-TKA differs significantly as it typically presents as an atraumatic pathology characterized by bone insufficiency in contact with the femoral component, hence its specific terminology [1-4].

Current literature suggests that various factors increase the risk of FCIF, including advanced age, obesity, female gender, osteoporosis, severe preoperative varus or valgus deformities, small femoral condyles, and the use of posterior-stabilized (PS) implants [1,3,4]. The postulated mechanism of failure involves overloading a preoperatively unloaded and osteopenic contralateral condyle [1].

In this retrospective review, we analyzed 835 primary total knee arthroplasties performed by a single surgeon and presented a series of six consecutive patients who developed atraumatic FCIF-TKA to evaluate its incidence, predisposing risk factors, management strategies, one-year outcomes, and up to four-year revision rates. Additionally, we reviewed the literature on FCIF-TKA and discussed strategies to mitigate its occurrence.

## Materials and methods

The SingHealth Centralised Institutional Review Board approved the study (approval number: 2024-3034) before the study commenced, ensuring compliance with ethical standards. This retrospective analysis reviewed postoperative radiographs and clinical documentation from 835 primary total knee arthroplasties performed between June 2018 and December 2023 by a single surgeon at the Department of Orthopaedic Surgery, Sengkang General Hospital, Singapore. Two independent authors identified cases of FCIF-TKA that occurred within six months postoperatively. FCIF-TKA was defined as a compression or impaction-type fracture of the femoral condyle occurring without trauma. Any discrepancies in case identification were resolved through discussion with a third independent author.

Patient demographic data included age, gender, BMI, past medical history, history of fragility fractures, and bone mineral density scans where available. Implant-specific data were collected, including femoral component design, polyethylene insert type, tibial component design, implant sizes, and whether stemmed components were used. Manufacturer specifications were reviewed to assess differences, such as chamfer cut size.

Preoperative radiographs, including weight-bearing anterior-posterior (AP), lateral, skyline, and long-leg alignment views, were examined to collect data on tibiofemoral angle and osteoarthritis grade, classified using the Kellgren-Lawrence scale (grades 0-4). The tibiofemoral angle was determined by drawing a line from the center of the proximal femoral shaft to the knee and another from the center of the tibial shaft distal to the knee. Postoperative AP, lateral, and skyline radiographs were analyzed for alignment and to determine the timing of FCIF-TKA diagnosis.

Range of motion (ROM) and weight-bearing visual analog scale (VAS) scores were recorded at four time points: immediately postoperatively, at the time of FCIF-TKA diagnosis, at six months, and at one year. Data on revision surgeries performed up to four years post-diagnosis were also collected.

Management of all cases initially involved the use of a hinged knee brace that allowed unrestricted ROM and toe-touch weight-bearing for the first six weeks. This was followed by partial weight-bearing (20 kg) for another six weeks, with full weight-bearing permitted at 12 weeks post-diagnosis.

## Results

**Figure 1:**
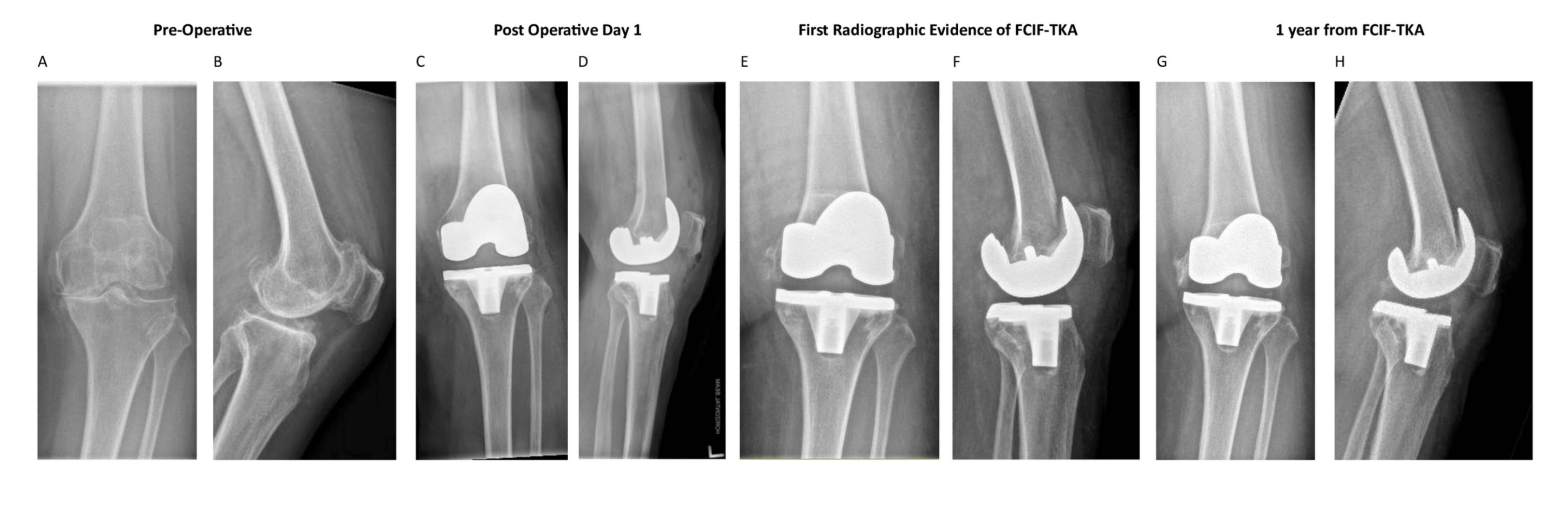
Patient 1 AP and lateral knee FCIF-TKA radiographs A: preoperative AP film, B: preoperative lateral film, C: postoperative day 1 AP film, D: postoperative day 1 lateral film, E: first radiographic evidence of FCIF-TKA AP film, F: first radiographic evidence of FCIF-TKA lateral film, G: one year from FCIF-TKA AP film, H: one year from FCIF-TKA lateral film AP: anterior-posterior, FCIF-TKA: femoral condyle insufficiency fractures following total knee arthroplasty

**Figure 2:**
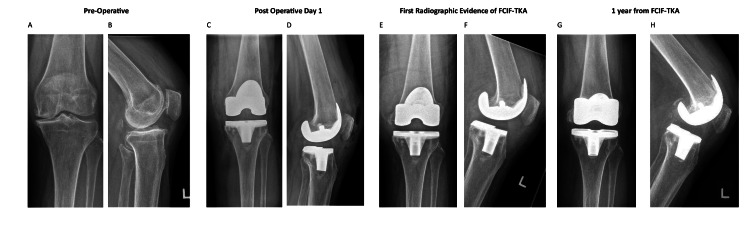
Patient 2 AP and lateral knee FCIF-TKA radiographs A: prepperative AP film, B: preoperative lateral film, C: postoperative day 1 AP film, D: postoperative day 1 lateral film, E: first radiographic evidence of FCIF-TKA AP film, F: frst radiographic evidence of FCIF-TKA lateral film, G.: one year from FCIF-TKA AP film, H: one year from FCIF-TKA lateral film AP: anterior-posterior, FCIF-TKA: femoral condyle insufficiency fractures following total knee arthroplasty

**Figure 3:**
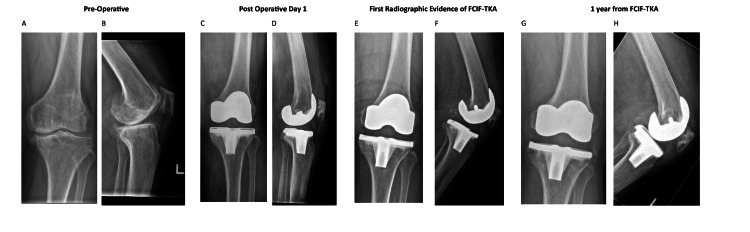
Patient 3 AP and lateral knee FCIF-TKA radiographs A: preoperative AP film, B: preoperative lateral film, C: postoperative day 1 AP film, D: postoperative day 1 lateral film, E: first radiographic evidence of FCIF-TKA AP film, F: first radiographic evidence of FCIF-TKA lateral film, G: one year from FCIF-TKA AP film, H: one year from FCIF-TKA lateral film AP: anterior-posterior, FCIF-TKA: femoral condyle insufficiency fractures following total knee arthroplasty

**Figure 4:**
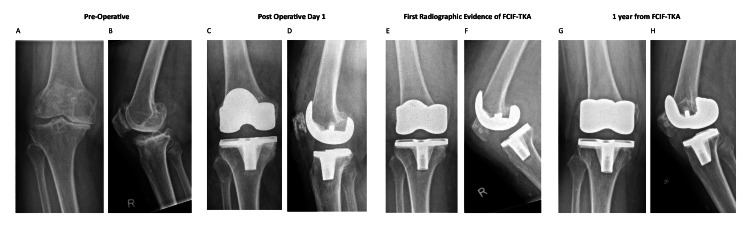
Patient 4 AP and lateral knee FCIF-TKA radiographs A: preoperative AP film, B: preoperative lateral film, C: postoperative day 1 AP film, D: postoperative day 1 lateral film, E: first radiographic evidence of FCIF-TKA AP film, F: first radiographic evidence of FCIF-TKA lateral film, G: one year from FCIF-TKA AP film, H: one year from FCIF-TKA lateral film

**Figure 5:**
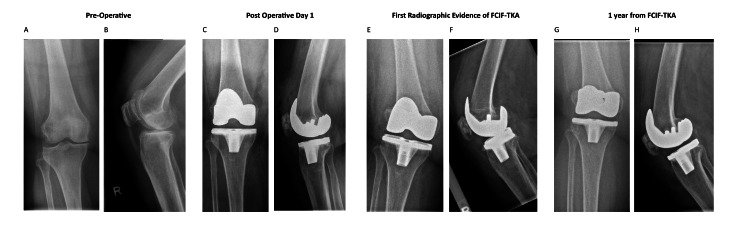
Patient 5 AP and lateral knee FCIF-TKA radiographs A: preoperative AP film, B: preoperative lateral film, C: postoperative day 1 AP film, D: postoperative day 1 lateral film, E: first radiographic evidence of FCIF-TKA AP film, F: first radiographic evidence of FCIF-TKA lateral film, G: one year from FCIF-TKA AP film, H: one year from FCIF-TKA lateral film AP: anterior-posterior, FCIF-TKA: femoral condyle insufficiency fractures following total knee arthroplasty

**Figure 6:**
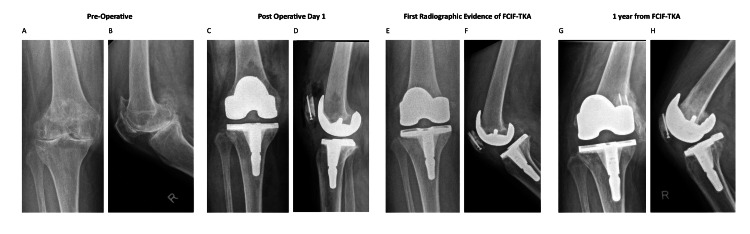
Patient 6 AP and lateral knee FCIF-TKA radiographs A: preoperative AP film, B: preoperative lateral film, C: postoperative day 1 AP film, D: postoperative day 1 lateral film, E: first radiographic evidence of FCIF-TKA AP film, F: first radiographic evidence of FCIF-TKA lateral film, G: one year from FCIF-TKA AP film, H: one year from FCIF-TKA lateral film AP: anterior-posterior, FCIF-TKA: femoral condyle insufficiency fractures following total knee arthroplasty

A review of 835 primary total knee arthroplasties identified an incidence of FCIF-TKA of 0.72% (six out of 835 cases), of which the radiographs from preoperative, postoperative day 1, day of FCIF-TKA diagnosis, and one-year post-FCIF-TKA are displayed in Figures 1-6. The mean age of affected patients was 72 years (range: 69-75), and the mean BMI was 33.48 (range: 25.3-39.3). Notably, all cases occurred in female patients. Bone mineral density scans were available for 83.3% (five out of six) of patients within one year of FCIF-TKA diagnosis, revealing osteopenia in 40% (two out of five) and osteoporosis in 60% (three out of five). Only one patient (16.7%) had a prior history of fragility fractures (Table 1).

**Table 1:** Patient demographics BMI: body mass index, VCFs: vertebral compression fractures

Demographics	Number	%
Age (years)	72 (69-75)	-
Female gender	6/6	100%
BMI, mean (range) (kg/m2)	33.48 (25.3-39.3)	-
Varus deformity	6/6	100%
Bone mineral density proven osteopenia	2/5	40%
Bone mineral density proven osteoporosis	3/5	60%
Previous fragility fracture, i.e., distal radius, proximal humerus, VCFs, or hip fracture	1/6	16.7%

The fractures were evenly distributed between the left and right sides, with 50% (three out of six) affecting each side. The mean tibiofemoral angle was 169.61° (range: 160-179.69°), with all cases presenting with varus deformity. The mean Kellgren-Lawrence grading was 4 for the medial compartment, 2.67 for the lateral compartment, and 3.5 for the patellofemoral compartment (Table 2).

**Table 2:** Preoperative measurements

Patient	Laterality	Tibiofemoral angle	Varus/valgus deformity	Medial compartment Kellgren-Lawrence grading	Lateral compartment Kellgren-Lawrence grading	Patellofemoral compartment Kellgren-Lawrence grading
1	Left	165.41°	Varus	4	2	4
2	Left	179.69°	Varus	4	3	3
3	Left	166.48°	Varus	4	3	3
4	Right	160.96°	Varus	4	2	4
5	Right	172.73°	Varus	4	2	3
6	Right	172.38°	Varus	4	4	4
Average	-	169.61°	-	4	2.67	3.5

All FCIF-TKA cases involved the use of Zimmer Persona PS cemented femoral implants (Zimmer Biomet, Warsaw, IN, USA). Implant sizes ranged from four to seven, and all were cemented. Fracture distribution was as follows: 67% (four out of six) were bicondylar, 17% (one out of six) involved the previously unloaded condyle, and 17% (one out of six) involved the previously loaded condyle. The mean time from surgery to FCIF-TKA diagnosis was 17.2 days (range: 14-21 days) (Table 3).

**Table 3:** Implant and FCIF-TKA details and clinical outcomes PS: posterior stabilized, FCIF-TKA: femoral condyle insufficiency fracture following total knee replacement, VAS: visual analog scale, ROM: range of motion, Y/N: yes/no, N/A: not available

Patient	Implant type and size		Time from surgery to fracture			Immediate post-op (VAS/ROM)	First diagnosis of FCIF-TKA (VAS/ROM)	6 months post-diagnosis of FCIF-TKA (VAS/ROM)	1 year post-diagnosis of FCIF-TKA (VAS/ROM)	Revision surgery performed
		Cement (Y/N)		Femoral condyle fracture affected (previously loaded, unloaded, both)	Compliance with brace					
1	1. Zimmer Persona PS size 4	Y	16 Days	Both	Yes	7, 10-95°,85°	5, 0-80°, 80°	2, 0-83°, 83°	1, 0-85°, 85°	No (@ 4 years)
	2. Insert 13 mm									
	3. Tibia size D									
2	1. Zimmer Persona PS size 7	Y	18 days	Both	Yes	2, 15-100°, 85°	5, 5-95°, 90°	0, 5-100°, 95°	0, 5-100°, 95°	No (@ 4 years)
	2. Insert 14 mm									
	3. Tibia size D									
3	1. Zimmer Persona PS size 5	Y	14 days	Both	Yes	5, 12-70°, 58°	5, 0-100°, 100°	2, 0-110°, 110°	1, 0-110°, 110°	No (@ 4 years)
	2. Insert 14 mm									
	3. Tibia size E									
4	1. Zimmer Persona PS size 7	Y	20 days	Both	Yes	2, 5-102°,97°	5, 5-95°, 90°	1, 5-100°, 95°	0, 5-100°, 95	No (@ 4 years)
	2. Insert 14 mm									
	3. Tibia size E									
	4. Patella 26 mm									
5	1. Zimmer Persona PS size 6	Y	21 days	Lateral	Yes	5, 17-100°, 83°	8, 15-95°, 80°	2, 0-80°, 80°	0, 0-100°, 100°	No (@ 4 years)
	2. Insert 11 mm									
	3. Tibia size D									
	4. Patella 26 mm x 7.5 mm									
6	1. Zimmer Persona PS size 7	Y	14 days	Medial	No	2, 10-60°, 50°	1, 3-95°, 92°	5, 0-70°, 70°	2, 0-80°, 80°	Yes (@ 13 months)
	2. Insert 12 mm									
	3. Tibia size D									
	Patella 29 mm x 8 mm									
Average	-	-	17.17 days	-	-	3.8, N/A, 76.3°	4.8, N/A, 88.7°	2.0, N/A, 88.8°	0.67, N/A, 94.2°	

All patients underwent blood tests at the time of diagnosis to rule out infectious causes. Management consisted of a hinged knee brace allowing unrestricted ROM and toe-touch weight-bearing for six weeks, followed by partial weight-bearing (20 kg) for an additional six weeks and full weight-bearing at 12 weeks post-diagnosis. Figure 1 illustrates the AP and lateral radiographs of the six patients up to one-year post-diagnosis.

Knee pain during weight-bearing was evaluated using VAS scores, recorded at four time points: immediately postoperatively (3.8), at diagnosis (4.8), at six months (2.0), and at one year (0.67). ROM showed progressive improvement, from an average of 76.3° immediately postoperatively to 88.7° at diagnosis, 88.8° at 6 months, and 94.2° at one year (Table 3).

**Figure 7:**
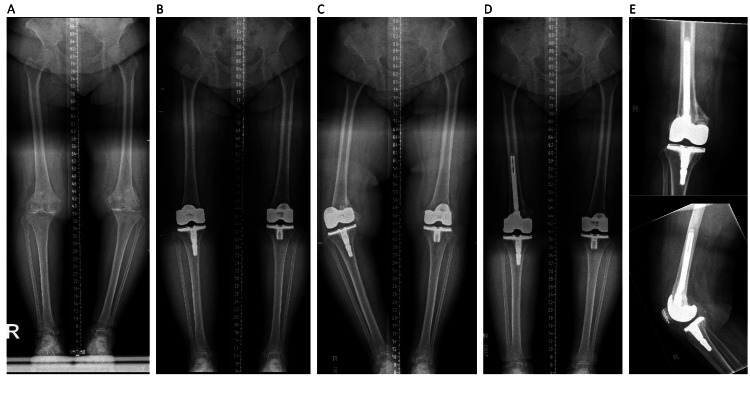
Patient 6 serial radiographs showing the alignment view and post-revision AP and lateral radiographs in chronological order from left to right A: preoperative long leg film, B: two weeks postoperative long leg film, C: one year post FCIF-TKA long leg film, D: two weeks post revision long leg film, E: two weeks post revision AP and lateral radiographs AP: anterior-posterior, FCIF-TKA: femoral condyle insufficiency fractures following total knee arthroplasty

Long-term follow-up showed that 83.3% (five out of six) of cases achieved implant survivorship without further revision at a minimum of four years. One patient (16.7%) required revision surgery due to non-compliance with the management protocol, resulting in fracture progression and collapse, as noted in serial radiographs. This patient underwent a revision to a stemmed femoral component with augmentation and a liner change at 13 months post-diagnosis (Table 3, Figures 6-7).

## Discussion

The underlying pathology and mechanism of FCIF-TKA differ significantly from traumatic periprosthetic fractures. FCIF-TKA is primarily associated with compressive loads on osteopenic bone, a factor compounded by pre-existing conditions such as varus/valgus deformities, osteoporosis, and high BMI [1-5]. Patient-specific risk factors include significant preoperative varus or valgus deformities, where alignment corrections during TKA can significantly alter biomechanical loading on the femoral condyles, predisposing patients to insufficiency fractures [1,3,4,6]. Additional systemic risk factors, such as age, female gender, smoking, and rheumatic diseases, further compromise bone quality [4,5,7,8]. Our results support this of this literature, with our six FCIF-TKA cases occurring in female, osteoporotic, and high BMI patients. An interesting point for discussion is the fracture location. Contrary to prior reports suggesting that FCIF-TKA primarily affects the previously unloaded condyle (e.g., lateral condyle in preoperative varus knees), our series predominantly found fractures involving the previously loaded condyle. This discrepancy indicates that current mechanisms explaining FCIF-TKA may need refinement. Understanding these nuances could lead to better-targeted preventive strategies [1].

The femoral component design in TKA has emerged as a possible factor in FCIF-TKA risk. Our series exclusively found FCIF-TKA in patients with PS implants, consistent with literature suggesting higher compression stress due to reduced metal bone contact in the weight-bearing zone of the femoral implant in PS-TKAs due to the greater amount of bony resection required for the box cut [1-4]. The role of femoral stems also requires attention; our findings aligned with existing reports that stemless implants present a higher risk of FCIF-TKA, possibly due to insufficient load distribution [1-4].

Management of FCIF-TKA is not extensively covered in the literature. Current case series often recommend revision surgery within six weeks of FCIF-TKA diagnosis [1-4]. However, our experience supports conservative management using a hinged knee brace for varus/valgus stability and a 12-week protected weight-bearing period. This approach showed promising outcomes, with compliant patients achieving good VAS and ROM scores and only one non-compliant patient requiring revision surgery at 13 months. This suggests that delayed revision surgery, after sufficient bony healing, can avoid extensive reconstructive procedures like distal femoral replacement [9,10]. Exploring management parallels with similar pathologies, such as vertebral compression fractures (VCFs), could offer insights. VCFs, like FCIF-TKA, are characterized by underlying bone insufficiency. Management techniques for VCFs, including subchondroplasty and the use of anti-osteoporotic medications, might be applicable to FCIF-TKA to enhance conservative treatment effectiveness. However, robust evidence supporting these interventions in FCIF-TKA is currently lacking and warrants further study.

To reduce FCIF-TKA incidence, especially in high-risk patients, specific preventive strategies can be considered. These include opting for cruciate retaining TKAs over PS TKAs, using femoral stems to improve load distribution, and employing robotic assistance to avoid intramedullary jigs or pins that might compromise bone integrity. Such strategies can increase bone-metal contact in the weight-bearing zone and safeguard osteoporotic bone.

Limitations

The limitations of this study include its retrospective design, which may introduce selection and recall biases. Additionally, the small sample size of six cases limits the generalisability of the findings and statistical analysis. All surgeries were performed by a single surgeon in a single institution, potentially restricting the applicability of results to broader populations. Lastly, while follow-up is extended to four years, longer-term outcomes and potential delayed complications remain unassessed.

## Conclusions

FCIF-TKA occurred in 0.72% of cases in our cohort, notably higher than the 0.05% reported in the existing literature. These fractures were associated with female patients with advanced age (mean 72 years), high BMI (mean 33.48), osteoporosis or osteopenia, and severe varus deformities, with FCIF-TKA only exclusively seen in stemless PS implants. Conservative management using a hinged knee brace and a 12-week progressive weight-bearing protocol significantly improved pain (mean VAS from 4.8 at diagnosis to 0.67 at one year) and ROM (from 88.7° at diagnosis to 94.2° at one year), with 83.3% survivorship at four years, which may suggest that immediate revision may not be necessary for compliant patients.
